# Associations of Physician Perspectives, Personal Choices, and Counseling for Severe Congenital Heart Defects

**DOI:** 10.1002/pd.6901

**Published:** 2025-09-23

**Authors:** Joyce L. Woo, Tara M. Swanson, Shiraz A. Maskatia, Shaine A. Morris, John D. Lantos, Angira Patel

**Affiliations:** 1 Ann & Robert H. Lurie Children's Hospital of Chicago, Northwestern University Feinberg School of Medicine, Chicago, Illinois, USA; 2 Kansas City Pediatric Cardiology Associates, Kansas City, Missouri, USA; 3 Lucile Packard Children's Hospital, Stanford University School of Medicine, Palo Alto, California, USA; 4 Baylor College of Medicine, Texas Children's Hospital, Houston, Texas, USA; 5 JDL Bioethics Consulting Group, Ossining, New York, USA

## Abstract

**Objective:**

To assess whether physicians' perspectives of outcomes or personal choices are associated with prenatal counseling for termination of pregnancy (TOP) or perinatal hospice for severe congenital heart defects (CHDs).

**Method:**

Multicenter survey of pediatric cardiologists and congenital heart surgeons in the United States, 2016–2018. The survey measured perspectives of CHD outcomes, personal choices/considerations, and prenatal counseling for TOP or perinatal hospice. Bivariate comparisons estimated associations between outcome perspectives, personal choices, and counseling.

**Results:**

Response rate was 77% (135/176); 47% (63/135) believed that single-ventricle portends good long-term quality of life or transplant-free survival. The majority (70%–90% depending on CHD type) would consider perinatal hospice for their own child, but a minority (2%–16% depending on CHD type) would choose perinatal hospice over TOP or postnatal intervention. Physicians who would consider TOP for themselves/their partner were more likely to counsel about TOP than physicians who would not consider TOP for themselves/their partner (99% vs. 67%, *p* < 0.001). There were no associations between institutional practice, outcome perspectives, personal consideration for hospice, and counseling practices.

**Conclusion:**

Physician perspectives of single-ventricle outcomes remain guarded but were not associated with counseling. However, personal choices/considerations are associated with counseling practices; therefore, considering personal beliefs is crucial to help families make fully informed decisions.

## 1 ∣ Introduction

Prenatal diagnosis of significant congenital heart disease (CHD), including single ventricle, allows for management decisions to be made during pregnancy, including termination of pregnancy (TOP), perinatal hospice (i.e., hospice birth plan, palliative birth plan, or comfort care without surgical intervention), or postnatal intervention (i.e., surgical/catheterization-based palliation or cardiac transplantation) [1, 2]. Prenatal diagnosis rates of single ventricle CHDs are high in the modern era (60%–70% [3, 4]), making prenatal counseling the first opportunity for families to receive comprehensive information about all three treatment options.

There is significant variability in whether all of these treatment options are discussed with parents during pediatric cardiologists' prenatal counseling. In national survey studies of physicians in North America, 64%–80% report counseling parents about TOP [5-8], whereas 56%–72% report discussing perinatal hospice [5-7, 9]. Reducing variability in prenatal counseling is essential to respect and empower families affected by fetal single ventricle disease [10, 11]. However, there remains a paucity of knowledge about the factors that drive variability in prenatal counseling practices.

To our knowledge, the only investigation on this topic within the United States was conducted by Haxel and Ronai et al., who found that physicians’ beliefs about life are associated with their TOP counseling for fetal hypoplastic left heart syndrome (HLHS) [7]. This analysis was conducted after the 2022 *Dobbs v. Jackson Women's Health Organization* (*Dobbs*) ruling, which revoked the constitutional right to termination and returned authority to regulate termination to individual states, thus impacting access to abortion care in certain geographic areas of the United States [12, 13]. The *Dobbs* ruling introduces further complexity in our understanding of the driving factors of prenatal CHD counseling.

We therefore expand on this knowledge through three objectives: first, we leverage an earlier multicenter national survey [14] to estimate associations between physicians' beliefs and counseling practices before the *Dobbs* ruling. Second, we report multiple dimensions of beliefs, including perspectives of clinical outcomes, and personal choices and considerations for TOP or perinatal hospice if one's own child had single ventricle disease. Third, we describe these beliefs across multiple types of single ventricular CHD, rather than HLHS alone.

## 2 ∣ Methods

### 2.1 ∣ Study Population

Pediatric cardiologists (i.e., all cardiac subspecalties) and cardiothoracic surgeons (physicians) from four medium-to-high volume CHD surgical centers across the United States (Ann & Robert H. Lurie Children's Hospital of Chicago, Chicago, Illinois; Lucile Packard Children's Hospital Stanford, Palo Alto, California; Children's Mercy Kansas City, Kansas City, Missouri; and Texas Children's Hospital, Houston, Texas; were surveyed between 2016 and 2018. Surveys were completed prior to the 2022 *Dobbs v. Jackson Women’s Health Organization* (Dobbs) ruling, which revoked the constitutional right to termination and returned authority to individual states [12, 13], thus eliminating any confounding or responder bias due to the *Dobbs* decision.

### 2.2 ∣ Survey Development and Distribution

The initial survey and iterative modifications were designed by fetal cardiologists (T.S., A.P.) and bioethicists (J.L., A.P.) based on previous studies evaluating *post*natal counseling practices for HLHS [15, 16]. Demographic questions were limited in the survey to maintain anonymity amongst respondents and maximize the survey response rate. Next, the survey was independently evaluated for feasibility and relevance by team members at the remaining CHD surgical centers (S.M., S.M.). Finally, the survey was sent to all cardiologists and surgeons at the four institutions. Three reminders were sent via email to maximize the response rate; there was no incentive provided for survey completion. All survey responses were collected with Research Electronic Data Capture (REDCap) [17] hosted at the Northwestern University Clinical and Translational Sciences Institute.

The final version of the survey contained a total of 60 questions. Of these, 23 questions pertained to physician support for patients' *post*natal decision-making, the subject of a previous analysis [14]. The remaining 37 questions pertained to physician perspectives, personal choices, and personal considerations and *pre*natal counseling practices, the subject of this analysis (Table S1). Specifically, we evaluate physicians' *perspectives* (i.e., their views based on clinical practice and existing literature) of outcomes, as well as their personal *choices and considerations* (i.e., what they would do or weigh if impacted by fetal CHD). These 37 questions were organized into three sections:

1. Physician perspectives and practices pertaining to prenatal counseling for severe fetal CHD. Respondents were given a five-point Likert scale to quantify their personal perspectives or counseling practices (Q1–20) as follows:
  1. “Completely agree”
  2. “Moderately agree”
  3. “Neither agree nor disagree”
  4. “Moderately disagree”
  5. “Completely disagree”
2. Physician *perspectives* of single ventricular outcomes (Q21–29). Responses were collected with the same five-point Likert scale as the first section.
3. Physicians' personal *choices and considerations* if their own fetus had single ventricle CHD. First, physicians were asked what their personal *choice* of management would be for four specific CHDs: HLHS with restrictive atrial septum, HLHS without restrictive atrial septum, pulmonary atresia with intact ventricular septum (PA/IVS), and tricuspid atresia (Q30–33, Section 3.1). The answer choices were as follows:
  1. “Termination”
  2. “Comfort care after birth”
  3. “Palliative Surgery”
  4. “Don't know, or undecided”

Next, physicians were asked about their personal *consideration for* termination or perinatal hospice if their fetus had significant CHD (Q34–37, Section 3.2), with “yes” and no” as the only options for response, without the option of “undecided”. For these four questions, we intentionally did not indicate a CHD type, as the “threshold” for TOP due to fetal CHD may individually differ (e.g., some might consider TOP for bicuspid aortic valve with aortic stenosis, while others might only consider TOP for single ventricle disease with heterotaxy).

### 2.3 ∣ Statistical Analysis

All data were deidentified prior to analysis. In descriptive analysis, responses for the first and second survey sections were collapsed into three categories: completely or moderately agree, neither agree or disagree, and completely or moderately disagree. We use the term “vast majority” to describe any question with ≥ 75% of responses, “majority” to describe any question with ≥ 50%, and “minority” to describe any question with < 50% of responses. To describe the degree of consensus in outcomes perspectives (Q21–29), we used a bar graph, ordering questions from the lowest to highest degree of agreement.

Specific survey questions were utilized to estimate associations between physicians' personal choices and counseling practices. Questions 21–29 measured physicians' perspectives of clinical outcomes, while Questions 34–37 measured physicians' personal consideration for TOP and/or perinatal hospice (Table S1 Question 8 (“Because of my religion/spirituality/morality, I do not support counseling a family about the option of termination of pregnancy.”) measured whether a physician would likely counsel about TOP in practice, where respondents answering Likert scores 1–3 were categorized as unlikely to counsel TOP, and those with Likert scores 4–5 categorized as likely to counsel TOP. Similarly, question 6 (“I would not mention perinatal hospice plan as an option for a serious congenital heart defect such as hypoplastic left heart syndrome, but if parents ask about this option I would discuss the benefits and burdens of this option with them.”) measured whether a physician would likely voluntarily counsel about perinatal hospice in practice, where Likert scores 1–3 were categorized as unlikely to voluntarily counsel and four to five likely to voluntarily counsel about perinatal hospice.

Therefore, to estimate associations between physicians' perspectives of outcomes and their likelihood of counseling TOP or perinatal hospice, Q21–24 and Q26–28, were compared with Q8 and Q6. To estimate associations between physicians' personal considerations for TOP or perinatal hospice and their likelihood of counseling TOP or perinatal hospice, Q34–37 were compared with Q8 and Q6. Bivariate comparisons were conducted with Chi-square or Fisher's exact tests, with a Bonferroni correction to adjust for multiple testing. To protect respondent privacy, no comparison testing was performed by demographic variables, or by physician type (cardiologist vs. surgeon). All analysis was completed in Stata18 (College Station, TX). This study was approved by institutional review boards from all four participating institutions.

## 3 ∣ Results

### 3.1 ∣ Respondent Characteristics and Counseling Practices

Of the 176 cardiologists and surgeons who were eligible for the survey, 77% (135/176) responded. Among respondents, 93% (126/135) identified as cardiologists and 7% (9/135) identified as cardiac surgeons. There were no incomplete surveys. There were no statistically significant differences in any of the responses by center. Gender and age data are shown in Table 1. Among respondents, 85% (Q8, 115/135) were likely to discuss TOP and 65% (Q6, 88/135) were likely to discuss perinatal hospice without patient prompting.

### 3.2 ∣ Physician Perspectives of Clinical Outcomes and Counseling Practices

**FIGURE 1 ∣:**
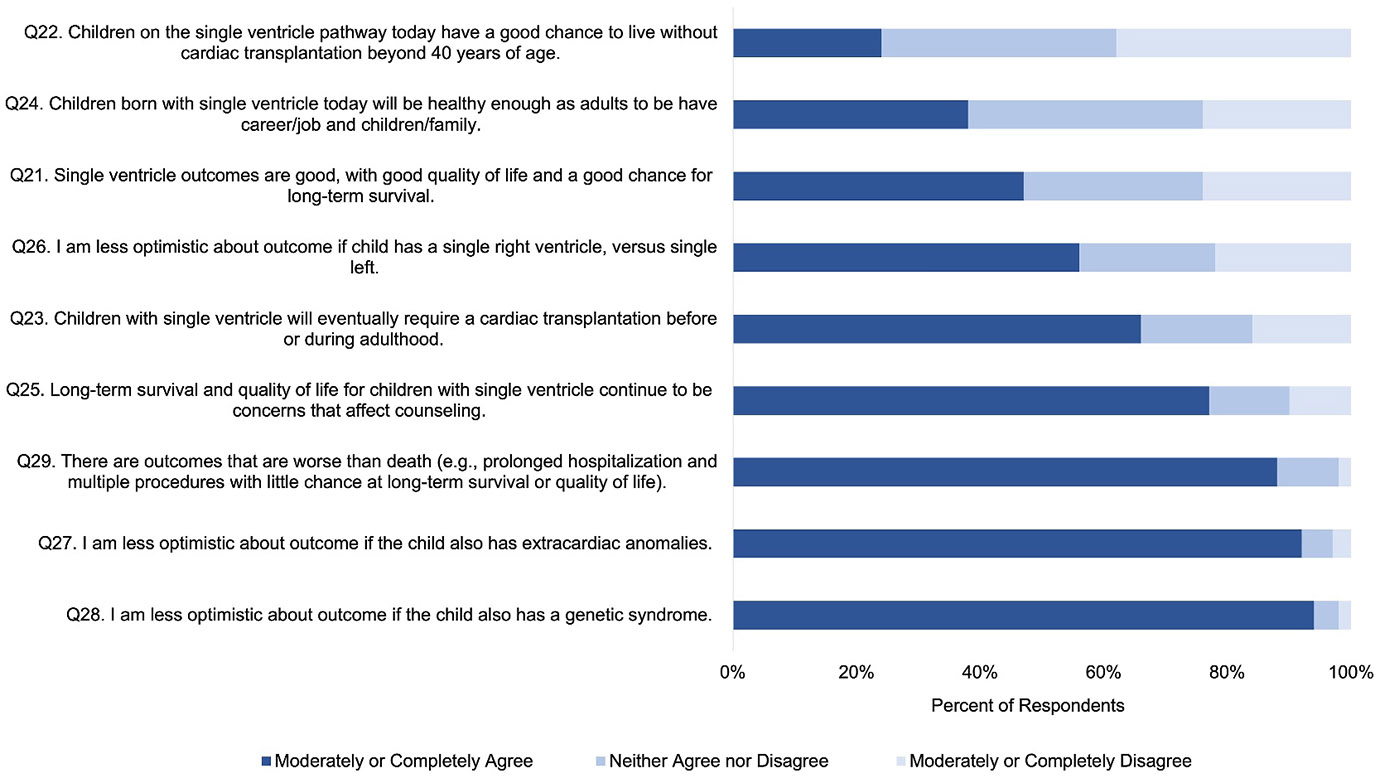
Physician perspectives on clinical outcomes of single ventricle congenital heart defects.

The vast majority of respondents (Q25, 77%, 104/135) agreed that long-term survival and quality of life were important aspects of prenatal counseling. The vast majority (Q29, 88%, 119/135) believed that some outcomes are worse than death for a child, including undergoing interventions with little chance at long-term survival or quality of life. Less than half (Q21, 47%, 63/135) of the respondents agreed that quality of life and long-term survival for children with a single ventricle are good. Specifically, a minority of respondents agreed that children born with a single ventricle today will be healthy enough to have a career/family (Q24, 38%, 51/135), and that children with a single ventricle have a good chance to live without transplantation beyond 40 years of age (Q22, 24%, 32/135, Figure 1).

The majority of respondents agreed that most children with a single ventricle will eventually require cardiac transplantation at some point (Q23, 66%, 89/135), and that a single left ventricle portends a better outcome than a single right ventricle (Q26, 56%, 76/135). There were no significant associations between physician perspectives of clinical outcomes (Q 21–24 and 26–28) and likelihood of counseling for TOP or perinatal hospice (Q8 and Q6).

### 3.3 ∣ Physicians' Personal Choices, Considerations, and Counseling Practices

**FIGURE 2 ∣:**
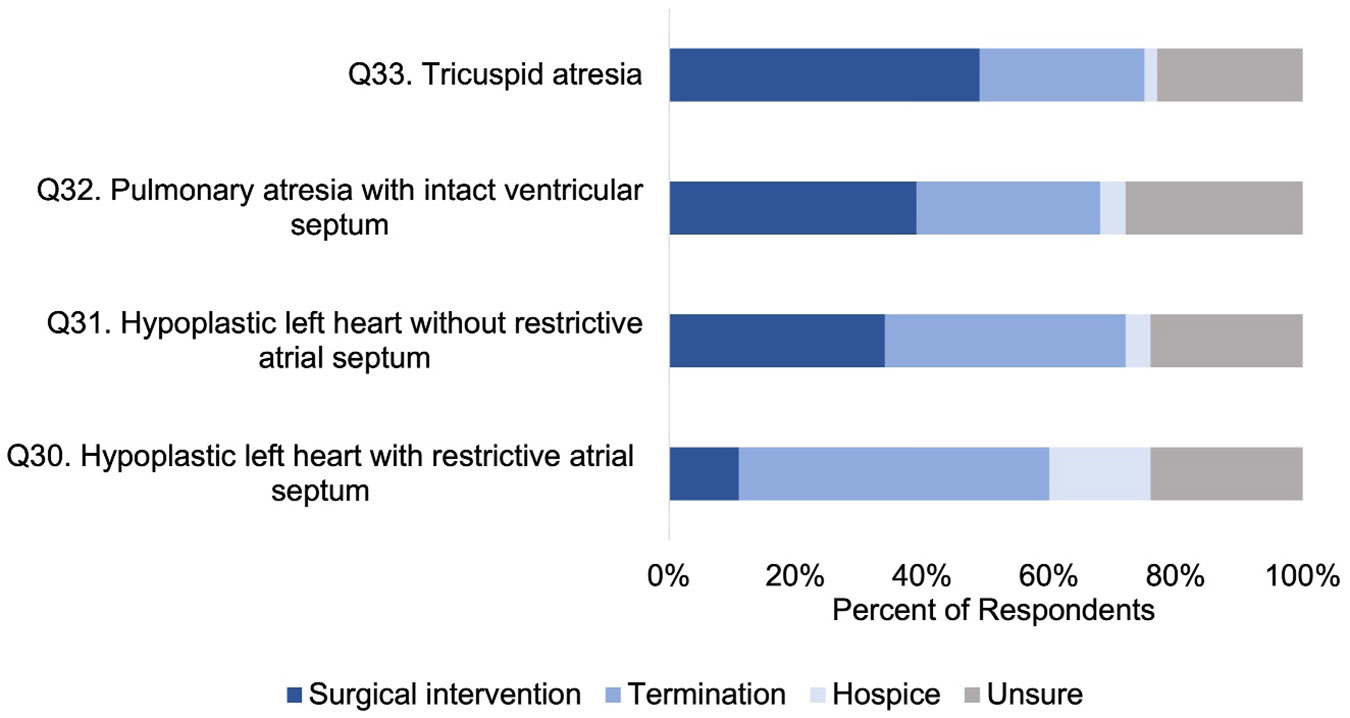
Physician *choices* in management if their own fetus was affected by single ventricular disease.

To elicit physicians' personal beliefs, we asked what management option physicians would *choose* as parents if their fetus was affected by different types of single ventricle CHD Responses varied by CHD type; 49% (Q33, 66/135) would choose postnatal surgical intervention for fetal tricuspid atresia, while 11% (Q30, 15/135) would choose postnatal surgical intervention for hypoplastic left heart syndrome with intact atrial septum. Respondents tended to choose termination of pregnancy if they opposed postnatal surgical intervention: 49% (Q30, 66/135) would choose TOP for hypoplastic left heart syndrome with intact atrial septum, whereas 26% (Q33, 35/135) would choose TOP for tricuspid atresia. A minority (Q30%–33%, 2%–16%, depending on CHD type) would choose perinatal hospice (Figure 2).

**FIGURE 3 ∣:**
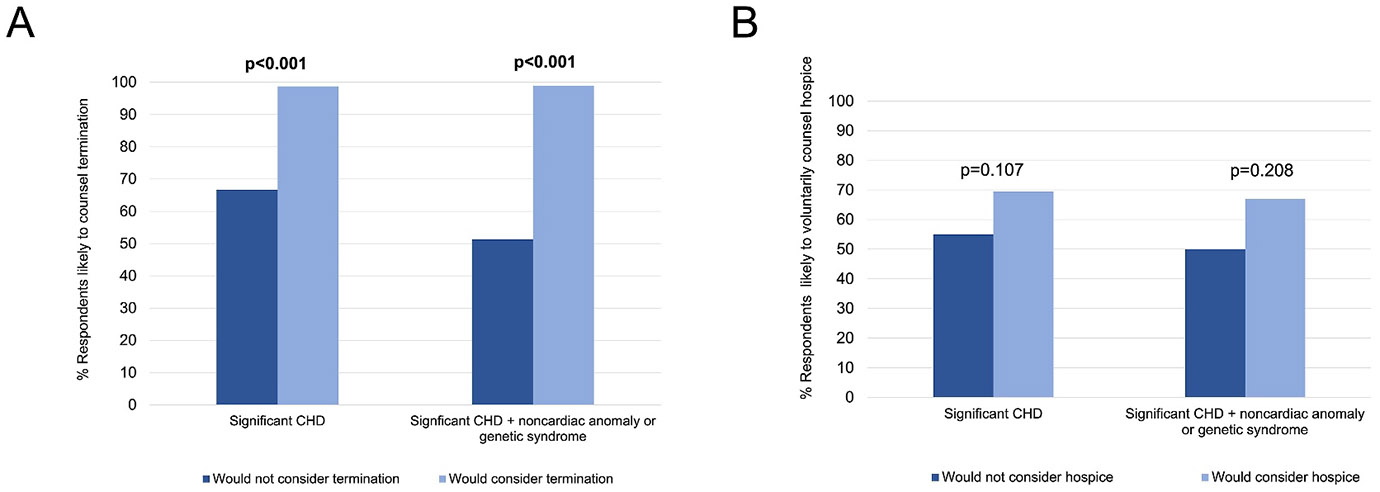
Associations between personal *considerations* and counseling practices for termination (A) or perinatal hospice (B).

The majority of those who would not personally consider TOP would still counsel TOP (67%, 38/57), while the vast majority for whom TOP was an option would counsel TOP (99%, 77/78, *p* < 0.001). This difference was more pronounced if their fetus were to have CHD with a noncardiac anomaly or genetic syndrome (51 vs. 99%, *p* < 0.001, Figure 3A). Whether hospice was a personal consideration was not associated with the likelihood of counseling hospice for patients (55% vs. 69%, *p* = 0.107, Figure 3B).

## 4 ∣ Discussion

In this multicenter, cross-sectional survey analysis of cardiologists and surgeons pertaining to their beliefs and counseling practices, we report four main findings. First, physician perceptions of long-term outcomes of single ventricle CHD remain guarded, with a minority (< 50%) of physicians perceiving good long-term quality of life or transplant-free survival for single ventricle CHD (Figure 1). Second, while a high number of respondents (70%–90%) would *consider* perinatal hospice for their own child, only a small minority of physicians, 2%–16% depending on CHD type, would *choose* perinatal hospice over TOP or surgical palliation (Figure 2). Third, personal considerations were more strongly associated with counseling practices for TOP than perinatal hospice (Figure 3), but this relationship was not absolute: 67% of those who would not personally choose TOP would still likely counsel TOP. Finally, perspectives of CHD outcomes were not associated with their counseling practices.

Few studies have examined physician perspectives of CHD outcomes [18], possibly because the establishment of measurable outcomes, beyond mortality and medical comorbidity, is a fairly recent development [19, 20]. We find that physicians' perspectives on outcomes remain guarded about both quality of life and life expectancy. These results echo those in previous studies that have shown that physicians tend to be less optimistic than parents of children with CHD [21, 22]. Whether physicians' guardedness is warranted depends on outlook. On one hand, the life expectancy for patients with severe CHD has dramatically increased since the late 20^th^ century due to rapid advancement in treatments, where the adult CHD population has now exceeded the pediatric CHD population in some higher-income countries [23]. On the other hand, when compared with the general population, adults with severe CHD still face significantly more psychosocial barriers [24, 25] and a shorter lifespan. In a Dutch longitudinal study, the estimated median survival was 53 years for those with severe CHD, versus 84 years in the general population [26]. This could be an overestimate because the observation period began at 18 years, thus excluding all earlier childhood CHD-related deaths. In a recent single-center analysis of HLHS, transplant-free survival was 31% at 35 years [27].

Regardless, we found no association between physicians' perspectives of outcomes and counseling practices. To our knowledge, while there is limited information on perspectives of outcomes in the current era, Kon et al. [15] studied *post*natal counseling practices for HLHS and similarly found that perspectives of surgical outcomes were not associated with postnatal recommendations for surgical intervention versus comfort care. Instead, Kon et al. found that institutional practices played a primary role in postnatal counseling for HLHS. In contrast, our study focused on prenatal counseling and found no significant institutional differences in TOP or perinatal hospice counseling. This suggests that unlike postnatal counseling, in the prenatal period, physicians' personal beliefs play a greater role in counseling variation, particularly for TOP, than institutional practice.

This analysis had several similar findings with Haxel and Ronai et al.'s recent study of fetal cardiologists' personal beliefs and their prenatal cardiac counseling [7], which was conducted in the first few months following the *Dobbs* ruling. Surprisingly, both analyses showed similar counseling rates for TOP (85% in this analysis, 80% in Haxel and Ronai et al.'s analysis, *p* = 0.28) and perinatal hospice (65% in this analysis, 72% in Haxel and Ronai et al.'s analysis, *p* = 0.27), and a significant association between personal beliefs and counseling practices for TOP. This suggests that at least in the immediate period following the *Dobbs* ruling, the relationship between beliefs and TOP counseling was similar.

However, a key difference between this analysis and Haxel and Ronai et al.'s was the association between beliefs and counseling for perinatal hospice. Haxel and Ronai et al. found that those who agree with “some life is better than no life at all” were less likely to value prenatal counseling for perinatal hospice (92% of those who agree/remain neutral with “some life is better than no life” also agreed with discussing a “comfort care” alternative, vs. 99% among those who disagreed with “some life is better than no life”, *p* = 0.04). In contrast, this analysis detected no association between personal consideration for perinatal hospice and perinatal hospice counseling. We hypothesize that the likely reason for this difference is that *value* for perinatal hospice and discussing perinatal hospice *in practice* are different. Indeed, in a multicenter survey evaluating pediatric cardiologists' attitudes about palliative care (prenatally or post-surgically), while 85% agreed that palliative care consultations are valuable, only 20%–50% of cardiologists felt very competent in discussing life expectancy and goals of care [9], suggesting that a lack of knowledge or familiarity with perinatal hospice are stronger driving factors than beliefs when it comes to counseling. While an association between perinatal hospice and counseling might have been detected with a higher number of respondents, such a finding would still suggest that the effect of personal beliefs on counseling is greater for TOP than perinatal hospice. Future studies should evaluate knowledge, rather than beliefs, as another driving factor for variation in perinatal hospice counseling. Given the decreasing accessibility of TOP in certain geographic areas, this knowledge is crucial not only for the CHD population but also for many life-limiting fetal conditions [28].

There are limitations to this analysis. First, generalizability is limited because only four congenital heart centers were surveyed; however, this also contributed to the relatively high survey response rate. Second, we did not survey obstetricians, neonatologists, or palliative care specialists who also play significant roles in counseling for TOP and perinatal hospice, though their more extensive training in perinatal counseling compared with cardiologists and surgeons may have confounded our findings. Third, our survey was conducted prior to the *Dobbs* versus *Jackson* ruling, though this allowed us to compare results with a recent study that was conducted after the *Dobbs* ruling [7]. To our knowledge, there are no previous studies evaluating physician beliefs and prenatal counseling behaviors from the pre-*Dobbs* era. Fourth, the *Dobbs* ruling uniquely applies to patients in the United States, where rates of termination are comparatively lower than many other high-income countries [29]. Fifth, our survey was insufficiently granular to delineate the driving mechanisms for perinatal hospice counseling, or the threshold of CHD severity at which a physician might choose to counsel or personally consider TOP/hospice.

## 5 ∣ Conclusion

Counseling encounters for single ventricle CHD are shifting from the postnatal to prenatal period [3, 30], making TOP and perinatal hospice counseling highly relevant to this high-risk population. Physicians' personal beliefs are associated with TOP counseling, while their perspectives of outcomes were not. Thus, consideration of personal biases in TOP counseling remains critical to reduce counseling variation. In the post-*Dobbs* era, TOP has become less accessible, making perinatal hospice the only non-surgical option depending on local policy. A better understanding of the drivers of variation in perinatal hospice counseling is crucial to maintain equitable outcomes.

## Supplementary Material

Supplementary Material **Table S1:** Survey Results.

Additional supporting information can be found online in the Supporting Information section.

## Figures and Tables

**TABLE 1 ∣:** Respondent characteristics (*N* = 135).^a^

	*n* (%)
Provider type	
Cardiologist	126 (92.6)
Surgeon	9 (6.6)
Age in years^b^	
≤ 40	44 (32.6)
41–50	33 (24.4)
51–60	17 (12.6)
≥ 61	12 (8.9)
Unknown	29 (21.5)
Gender^b^	
Female	60 (44.4)
Male	46 (34.1)
Unknown	29 (21.5)
Institution number	
1	29 (21.5)
2	38 (28.1)
3	25 (18.5)
4	43 (31.9)

a Comparison testing by respondent characteristics was not performed to protect respondent privacy.

b Gender and age data were available for 3 of the 4 institutions, or 79% of respondents (*n* = 106).

## Data Availability

The data that supports the findings of this study are available in the supplementary material of this article.

## Summary

- What is already known about this topic?
  - Pediatric cardiologists' beliefs about life impact their prenatal discussions about termination of pregnancy due to fetal congenital heart defects.
- What does this study add?
  - Pediatric cardiologists' and congenital heart surgeons' perspectives on the long-term outcomes of severe congenital heart defects are guarded. However, these perspectives, and institutional practices, are not associated with prenatal counseling practices for termination or perinatal hospice, whereas personal choices and considerations are.
