# Supplementary material for: Associations of Physician Perspectives, Personal Choices, and Counseling for Severe Congenital Heart Defects
Source: Prenat Diagn. Author manuscript; Available in PMC 2025 Oct 20. (PMC12530347; doi:10.1002/pd.6901)
Supplement: Supplementary Material — Table S1: Survey Results. [file NIHMS2114034-supplement-Supplementary_Material.docx]

**Supplementary Material for *Associations of Physician Perspectives, Personal Choices, and Counseling for Severe Congenital Heart Defects.***

**Supplemental Table 1**. **Survey Results**

| Topic 1: Physician Perspectives of Counseling for Fetal CHD | | | | |
| --- | --- | --- | --- | --- |
| Survey Question | | Moderately or Completely Agree  n (%) | Neither Agree nor Disagree  n (%) | Moderately or Completely Disagree  n (%) |
| Q1. Families should be counseled about all reasonable management options when their child is newly-diagnosed with a congenital heart disease. | | 135 (100) | 0 (0) | 0 (0) |
| Q2. Discussion of the benefits and difficulties of each management option should be part of counseling the family of a child with newly-diagnosed congenital heart disease. | | 134 (99) | 0 (0) | 1 (1) |
| Q3. If more than one cardiologist/surgeon within our group would choose a management option for their own child, then that option is a "reasonable" choice. | | 103 (76) | 17 (13) | 15 (11) |
| Q4. Even if I believe a family should not choose a management option, I would still be able to present that option to parents if our group believes it is a "reasonable" option. | | 128 (95) | 1 (1) | 6 (4) |
| Q5. All options offered to prenatally-diagnosed families should also be offered to postnatally-diagnosed families (with the exclusion of termination of pregnancy). | | 103 (76) | 15 (11) | 17 (13) |
| Q6. I would not mention perinatal hospice plan as an option for a serious congenital heart defect such as HLHS, but if parents ask about this option I would discuss the benefits and burdens of this option with them. | | 34 (25) | 13 (10) | 88 (65) |
| Q7. Adoption is a realistic option for a newborn with a single-ventricle cardiac defect. | | 66 (49) | 36 (27) | 32 (24) |
| Q8. Because of my religion/spirituality/morality, I do not support counseling a family about the option of termination of pregnancy. | | 13 (10) | 7 (5) | 115 (85) |
| Q9. I do not feel comfortable counseling about the option of termination of pregnancy. | | 24 (18) | 9 (7) | 101 (75) |
| Q10. Because of my religion/spirituality/morality, I do not support counseling a family about the option of perinatal hospice plan. | | 3 (2) | 3 (2) | 129 (96) |
| Q11. I do not feel comfortable counseling about the option of perinatal hospice plan after birth. | | 3 (2) | 4 (3) | 123 (91) |
| Q12. Termination of pregnancy is ethically/morally/spiritually different than providing a perinatal hospice plan | | 39 (29) | 22 (16) | 74 (55) |
| Q13. The impact of a child with significant medical needs on the family unit (e.g. parental divorce rate, financial burden on a family, positive and negative impact on siblings) is a factor which should be openly discussed with families during counseling. | | 121 (90) | 9 (7) | 3 (4) |
| Q14. The impact of a child with significant medical needs on the family unit is an important factor that influences my counseling. | | 81 (60) | 31 (23) | 23 (17) |
| Q15. We should always act on what is in a child's best interest without considering the impact of a child's diagnosis on the family unit. | | 21 (28) | 20 (27) | 59 (80) |
| Q16. If we feel we have done our best to fully inform/educate a family, then we must support the decision the family makes regarding their child's care. | | 115 (85) | 13 (10) | 7 (5) |
| Q17. It is never possible to fully inform/educate a family. | | 80 (59) | 18 (13) | 38 (28) |
| Q18. We should add our medical opinion/expertise after describing each of the management options to help the family make a decision regarding their child's care. | | 115 (85) | 8 (6) | 13 (10) |
| Q19. Our institution and personnel are committed to doing everything possible to help children live. If parents choose perinatal hospice plan for their child, it would be best if they deliver at their small regional hospital and not come to our institution for postnatal care. | | 13 (10) | 13 (10) | 111 (82) |
| Q20. Our institution and personnel are committed to providing the best support for families and children, regardless of how little or how much medical treatment they want to pursue. If parents choose perinatal hospice plan for their child, they should deliver at our institution because we can provide the best support possible. | | 99 (73) | 23 (17) | 13 (10) |
| Topic 2: Physician Perspectives of CHD Outcomes | | | | |
| Survey Question | | Moderately or Completely Agree  n (%) | Neither Agree nor Disagree  n (%) | Moderately or Completely Disagree  n (%) |
| Q21. In general, the outcomes for children going down the single-ventricle palliative pathway are very good now, with usually a good quality of life and a good chance for long-term survival. | | 63 (47) | 40 (29) | 32 (24) |
| Q22. Children going down the single-ventricle palliative pathway today have a good chance to live (without cardiac transplantation) to 40 years old and beyond. | | 32 (24) | 51 (38) | 51 (38) |
| Q23. Most 'single ventricle' children will eventually require a cardiac transplantation during adulthood (if not before). | | 89 (66) | 24 (18) | 22 (16) |
| Q24. Most 'single ventricle' children born today will likely be healthy enough as adults to have a career/job and their own children/family if they desire. | | 51 (38) | 51 (38) | 32 (24) |
| Q25. Longer-term survival and quality of life for 'single ventricle' children continue to be significant concerns that affect my counseling. | | 104 (77) | 18 (13) | 13 (10) |
| Q26. I am much less optimistic about a child's outcome if he/she also has a single right ventricle (rather than a single left ventricle). | | 76 (56) | 30 (22) | 30 (22) |
| Q27. I am much less optimistic about a child's outcome if he/she also has extracardiac anomalies. | | 124 (92) | 7 (5) | 4 (3) |
| Q28. I am much less optimistic about a child's outcome if he/she also has a "genetic syndrome”. | | 127 (94) | 5 (4) | 3 (2) |
| Q29. There are some outcomes that are worse than death for a child, including prolonged hospitalization and multiple invasive procedures with little chance at long-term survival or quality of life. | | 119 (88) | 13 (10) | 3 (2) |
| Topic 3.1: Physicians’ Personal **Choices** of Management | | | | |
| Survey Question | Termination of Pregnancy  n (%) | Perinatal hospice plan  n (%) | Palliative Surgery  n (%) | Don’t know, or undecided  n (%) |
| Q30. If your child was prenatally diagnosed with HLHS and intact atrial septum, which management option would you and your spouse choose? | 66 (49) | 21 (16) | 15 (11) | 33 (24) |
| Q31. If your child was prenatally diagnosed with 'garden-variety' HLHS, which management option would you and your spouse choose? | 51 (38) | 6 (4) | 46 (34) | 32 (24) |
| Q32. If your child was prenatally diagnosed with PA/IVS, which management option would you and your spouse choose? | 39 (29) | 5 (4) | 53 (39) | 38 (28) |
| Q33. If your child was prenatally diagnosed with tricuspid atresia, which management option would you and your spouse choose? | 35 (26) | 3 (2) | 66 (49) | 31 (23) |
| Topic 3.2: Physicians’ Personal **Considerations** for Management | | | | |
| Survey Question | No  n (%) | | Yes  n (%) | |
| Q34. If your child was prenatally diagnosed with an isolated significant heart defect, would termination of pregnancy ever be an option for you and your spouse? | 57 (42) | | 78 (58) | |
| Q35. If your child was prenatally diagnosed with a significant heart defect as well as extracardiac anomalies and/or a likely genetic syndrome, would termination of pregnancy ever be an option for you and your spouse? | 39 (29) | | 96 (71) | |
| Q36. If your child was diagnosed with an isolated significant heart defect, would perinatal hospice plan ever be an option for you and your spouse? | 40 (30) | | 95 (70) | |
| Q37. If your child was diagnosed with a significant heart defect as well as extracardiac anomalies and/or a likely genetic syndrome, would perinatal hospice plan ever be an option for you and your spouse? | 14 (10) | | 121 (90) | |
